# A pilot study of red complex and three genera subgingival microbiome in periodontitis subjects with and without diabetes, evaluated by MinION platform

**DOI:** 10.12688/f1000research.28216.4

**Published:** 2021-07-12

**Authors:** Boy M. Bachtiar, Citra F. Theodorea, Dicky L. Tahapary, Cindy Astrella, Natalina -, Endang W. Bachtiar

**Affiliations:** 1 Department of Oral Biology and Oral Science Research Center, Faculty of Dentistry, Universitas Indonesia, Jakarta, 10430, Indonesia; 2 Division of Endocrinology, Department of Internal Medicine, Dr. Cipto Mangunkusumo National Referral Hospital, Faculty of Medicine, Universitas Indonesia, Jakarta, 10430, Indonesia; 3 Metabolic, Cardiovascular and Aging Cluster, The Indonesian Medical Education and Research Institute, Faculty of Medicine, Universitas Indonesia, Jakarta, 10430, Indonesia; 4 Department of Internal Medicine, Dr. Cipto Mangunkusumo National Referral Hospital, Faculty of Medicine, Universitas Indonesia, Jakarta, 10430, Indonesia; 5 Department of Periodontology, Faculty of Dentistry, Universitas Indonesia, Jakarta, 10430, Indonesia

**Keywords:** MinION, 16S rRNA, Red Complex bacteria, Diabetes, Periodontitis, Subgingival Microbiome

## Abstract

**Background**: Subgingival niche is one biofilm habitat containing rich microbiota, which plays an active role in maintaining the health of periodontal tissue and determining host response. As such, a study of changing subgingival biofilms is important for understanding the effect of a systemic condition. In this study, we compared the occurrence of six bacteria cohabiting in the subgingival area of periodontitis subjects, with (DP, n = 8) and without (NDP, n = 4) diabetes.

**Methods**: The six genus and species of targeted bacteria were confirmed by 16S rRNA amplicon sequencing on MinION nanopore platform. Descriptive statistic was used to describe the obtained data.

**Results**: We found that the six genus and species of targeted bacteria were detected but in different quantities in either group's periodontal pocket. Our data showed that Tannerella forsythia was the most abundant species in subgingival biofilms of the DP group of the red complex bacteria. In contrast, Aggregatibacter sp., which belongs to the phylum of proteobacteria, was present at a relatively lower level. In contrast, Fusobacterium sp., which belongs to orange complex bacteria, showed relative similarities in subgingival biofilms of both groups tested, while Veillonella sp., were abundant in the DP groups.

**Conclusions: **Our data show that the diversity of classic periodontopathogens increased in the subgingival niche of periodontitis subjects with diabetes. It is the first study in Indonesia to apply MinION-based, full-length 16S rRNA amplicon sequencing in periodontitis patients with and without diabetes.

## Introduction

Taking DNA straight from oral samples, without culture samples, is a fundamental principle of oral microbiome study. Currently used molecular methods generally rely on PCR, which can be used to target specific bacterial species. However, detection is only enabled for those that have primers. PCR can also detect all bacterial species using a broad range of 16S primers followed by sequencing, but problems can arise from contamination ^1^.

The advancement of sequencing technology has led to the mainstream in oral microbiology due to the increasing affordability and improvement in the speed of the sequencing process and quality of data obtained. In this context, MinION is the first sequencer available commercially that uses nanopore technology. Unlike other sequencing technology, the sequencing method provided by the MinION device does not rely on the synthesis of nucleotides. For this reason, we decide to use this long-read nanopore sequencing device to identify the dominant bacteria in diabetic condition-related periodontitis.

One of the systemic conditions that have been asserted to affect the host immune response to dental plaque is diabetes ^2, 3^. However, the effect of diabetes on the composition of the subgingival microbiome is still inconclusive ^4^. A previous study showed that an increased number of periodontal pathogens had been isolated from the subgingival plaque of diabetic patients ^5^. However, another study showed a decrease of bacterial diversity associated with periodontitis ^6^, indicating a putative role of specific oral bacteria species in the oral niche that might be correlated to the condition due to diabetes ^7^.

Among the subgingival microbial community of periodontitis, the red complex bacteria ( *Phorphyromonas gingivalis*, *Treponema denticola*, and *Tannerella forsythia*) have been considered as major periodontal pathogens ^8, 9^. Their presence in the subgingival environment indicates that a selected bacteria species occurs due to the suitable anaerobic microenvironment that has been formed ^10^. However, these red complex bacteria are usually preceded by members of other complex oral bacteria species, including those associated with a healthy periodontal pocket ^11^. This indicates that ecological stress has occurred, leading to the presence of periodontitis-related microorganisms leading to an imbalance of bacteria species in the dental plaque ^10^. Consequently, in subgingival samples, the red complex bacteria could be measured as multiple oral bacteria species due to their role in progressive periodontitis.

The main goal of the current study was to use MinION of the full-length 16S rRNA gene to compare the profile of red complex bacteria and three other genera ( *Aggregatibacter, Fusobacterium*, *and Veillonella*) in two different subgingival niches of periodontitis, subjects with (DP) and without (NDP) diabetes, in order to assess their microbiome. 

## Methods

### Patients

This study was conducted between November 2018 and early June 2019. Twelve patients, 20 to 50 years of age, from consecutive participants were recruited from the Department of Internal Medicine, Faculty of Medicine, Universitas Indonesia, Cipto Mangunkusumo Hospital (FKUI-RSCM), Jakarta, Indonesia. The Ethics Committee of FKUI-RSCM has approved this study's protocol (No.1062/UN@.F1/ETIK/2018). The investigation procedure has been conducted according to the Declaration of Helsinki. Written informed consent was obtained from each subject to participate in this study.

Subjects excluded from this study were those who (i) had systemic disease other than diabetes mellitus; (ii) had received periodontal treatment or had taken antibiotic within the previous three months; (iii) and were smokers or pregnant. Participants in this study were those with periodontitis complicated by diabetes (DP, n = 8) and non-diabetes (NDP, n = 4), and diabetes criteria were determined from their medical record.

All patients consented to provide a subgingival dental plaque for this study. All subjects were diagnosed for periodontitis or healthy periodontal tissue according to criteria described by the American Academy of Periodontology ^12^. Subgingival dental plaque was collected by a registered dentist using sterile periodontal scalers and placed in individual microcentrifuge tube containing phosphate-buffered saline (PBS, pH 7.4). Samples were stored at -20°C until further processing.

The effects and efficiency of pooling samples have been investigated in many studies ^13, 14^. Therefore, in this study, we analysed pooled samples of subgingival biofilm of the two group patients with periodontitis for shifts in the subgingival community in response to a diabetic condition.

### Microbial sample

The samples were taken from three sites in DP subjects and three periodontal pockets in NDP subjects. Bacterial subgingival biofilm was collected from one diseased site (when present) with probing depth ≥5 mm with bleeding on probing. The collection area was isolated with cotton rolls, and a supragingival plaque was carefully removed with curettes. The collection was done with a sterile endodontic paper point by inserting the point to the depth of the sulcus and moving it laterally along the tooth's axis. Immediately following sampling, the paper point was placed in a microcentrifuge tube and stored at -70°C until processed.

In this study, the presence at genus and species level of the red complex bacteria ( *Phorphyromonas gingivalis, Treponema denticola*, and *Tannerella forsythia*), and three other selected genera ( *Aggregatibacter, Fusobacterium*, and *Veillonella*) were determined in subgingival biofilm samples. Therefore, we first extracted the DNA from the samples by using a DNA extraction kit Qiagen Q1Amp1 DNA Mini Kit, as per the manufacturer’s instruction. The obtained DNA’s concentration and quality were further determined using Qubit assay reagents (Invitrogen; Carlsbad, CA, USA). After dissolving in Tris-EDTA buffer, the DNA was cooled to -20°C until further processing.

### MinION sequencing and data analysis

The purified DNA were amplified by polymerase chain reaction (PCR) using specific primers (27F and 1492R) in a commercially available kit (16S Barcoding kit;SQK-RAB204; Oxford Nanopore Technologies/ONT, Oxford, UK). The procedure was conducted according to the protocol provided by 16S Barcoding Kit (SQK-RAB204; Oxford Nanopore Technologies/ONT, Oxford, UK). All PCR products for each subgingival biofilm sample obtained from their respective groups (DP and NDP) were pooled and purified. The concentration of each purified pool was measured using the Qubit dsDNA HS Kits by the Qubit Fluorometer (Invitrogen). For each purified pool, library preparation was prepared using the 16S Barcoding Kit mentioned above.

Two sequencing libraries were further prepared, one for a sample from DP and one for a sample from NDP. The amount of initial DNA used for both barcoding kits was 100 ng. Finally, each sequencing library was loaded into MinION flow cell of the MinION sequencing device (ONT) to be sequenced for 48 hours. Subsequently, the base calling of the generated data (fastq format) was analysed by using EPI2ME Desktop Agent (ONT). For microbiota profiling analysis, we followed the EPI2ME platform by selecting a workflow of 16S alignment for real time analysis. Alternatively, the obtained data can be analysed by a freely available software, NanoPipe (RRID:SCR_016852) ( www.bioinformatics.uni-muenster.de/tools/nanopipe2). Since the base calling process is the central to improving the accuracy of nanopore sequencing technology ^15^, in this study, only reads designated as ‘pass’ were included for further analyses. The analysis results were further generated in the form of a report in the EPI2ME platform.

### Data analyses

Descriptive analyses were performed with GraphPad Prism 9.0 (GraphPad Software, Inc., San Diego, CA). If the six bacteria were detected in the two sample groups, the group was regarded as positive for these bacteria.

## Results and discussion

The ONT has been reported for its potential benefits to analyse microbial communities' composition and dynamics, including oral pathogen ^16^. In this pilot study, we provide a new information on the state of ONTs MinION device for whole genome sequencing of some periodontal bacterial organisms. To describe the main finding of the results, we used the online EPI2ME platform, which contains a 16S workflow for analysing MinION reads.

### Read analysis

We endeavoured to determine if the oral microbial community would reveal the different profiles of the six selected periopathogens in pooled samples collected from periodontitis subjects with (DP) and without (NPD) diabetes. We used plaque subgingival biofilm samples for practical and economic reasons, which have often been employed ^17^.

**Figure 1.**
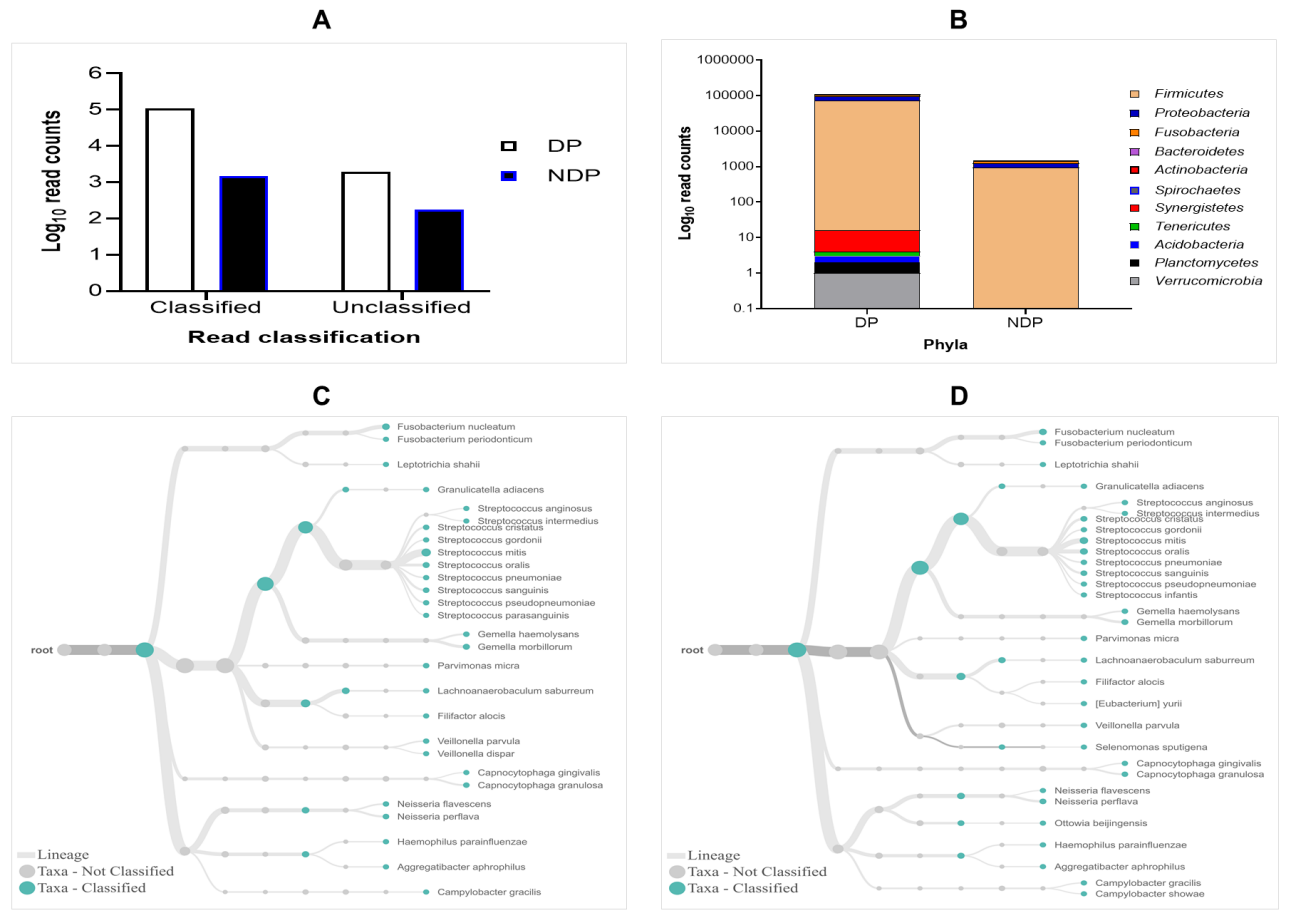
Read classification across pooled subgingival microbiota samples collected from diabetic (DP) and non-diabetic (NDP) patients. ( **A**) Taxa-level; ( **B**) phylum; ( **C** and **D**) root taxa in DP ( **C**) and NDP ( **D**) groups.

The results of 16S rRNA amplicons on MiniION sequencing revealed a total of 113,654 sequence reads after base-calling, with more reads classified than unclassified in either group. However, the subgingival biofilms obtained from the DP group were found better classified and a greater number of species compared to those found in the subgingival microbiome of the NDP group. By comparing the read count, we found that the classified and unclassified sequence reads in the pooled sample of DP were 112.173 and 1988, respectively, while the classified and unclassified sequence reads in the pooled sample of NDP were 1478 and 172, respectively ( Figure 1A). We found that the accuracy in pooled DP was 87% compared to those in pooled NDP that showed 85%. These results indicate that the long-read amplicons for sequencing on ONT covered nearly the full length of V1-V9 hypervariable regions of the 16S rRNA gene. In this study, all the reads were 1-directional base-calling, representing a sequence in the forward or reverse direction. Thus, the application of 16S rRNA-based using MinION platform has allowed tracking of bacterial cells' identity in subgingival niches, as shown in this study.

### Bacterial diversity and structure of the subgingival samples of periodontitis subjects, with and without diabetes

In the current study, the long length sequences were taxonomically assigned using a workflow from the cloud-based EPI2ME, which supported the NCBI 16S database, allowing classification down to the phylum, genus and species level, respectively. Therefore, we searched which taxa were responsible for overall community differences between DP and NDP groups. Our data showed that in general, subgingival microbiome patterns were similar between DP and NDP groups, a phenomenon that have been reported by ^18, 19^. Other studies ^20^ showed differences between glycaemic status and the proportion of several phyla. This study did not separate the tested sample according to the glycaemic level in diabetic subjects. We aimed to examine whether diabetes mellitus might affect composition of six targeted bacterial communities in the subgingival niche. Thus, we observed these subgingival bacteria by phylum to species level.

The obtained sequences were first analysed on taxonomic basis at the phylum level. Eleven phyla (Firmicutes, Proteobacteria, Fusobacteria, Bacteroides, Actinobacteria, Spirochaetes, Synergistetes, Tenericutes, Acidobacteria, Planctomycetes, and Verrucomicrobia) were detected in DP group, while the last five phyla were not detected in NDP group. This finding indicates that the ONT technology allowed sequencing of the entire 16S rRNA gene region of the bacteria belonging to these phyla associated with the disease processes in our diabetic patients. This result agrees with previous findings in the subgingival bacterial microbiome diversity study in diabetic patients ^21^. Interestingly, the long sequence of Synergistetes, which were only found in the DP group, has been identified in the area of periodontitis ^22^. The colonization are located in the outermost region of subgingival biofilm, indicating they are opposed to inflammatory cells ^23^. However, no genus belonging to Synergistic phylum was detected in this study. We assumed this is because of the low-read accuracy by MinION platform ^24^, which complicates our complex samples' analysis.

### Distribution of the subgingival microbiota at the phylum and family level

In this study, the subgingival microorganisms' samples were collected from the periodontal pocket at the same depth (≥ 5mm). Firstly, we found that the seven major phyla detected in subgingival microbiota of DP group were Firmicutes, Proteobacteria, Fusobacteria, Bacteroidetes, Actinobacteria, Spirochaetes, and Synergistetes, contain 99% of the taxa. The remaining phyla, Tenericutes, Acidobacteria, Planctomycetes and Verrucomicrobia containing the remaining 1% of the Taxa. Compared to the subgingival microbiota in the NDP group, containing four major phyla (99%): Firmicutes, Proteobacteria, Fusobacteria, and Bacteroides, while the Actinobacteria was the remaining bacteria containing 1% in NDP group. At the phyla level, the phyla occurrences belong to the six targeted bacteria in both groups are the same, but the proportions differed. The Firmicutes phylum dominated the microbial community with relative abundance higher than 80% in both pooled samples ( Figure 1B). This result demonstrated that using MinION, it is possible to associate the single cell level for nearly all subgingival plaque bacteria, from each group tested, to one of the major taxonomic units.

**Figure 2.**
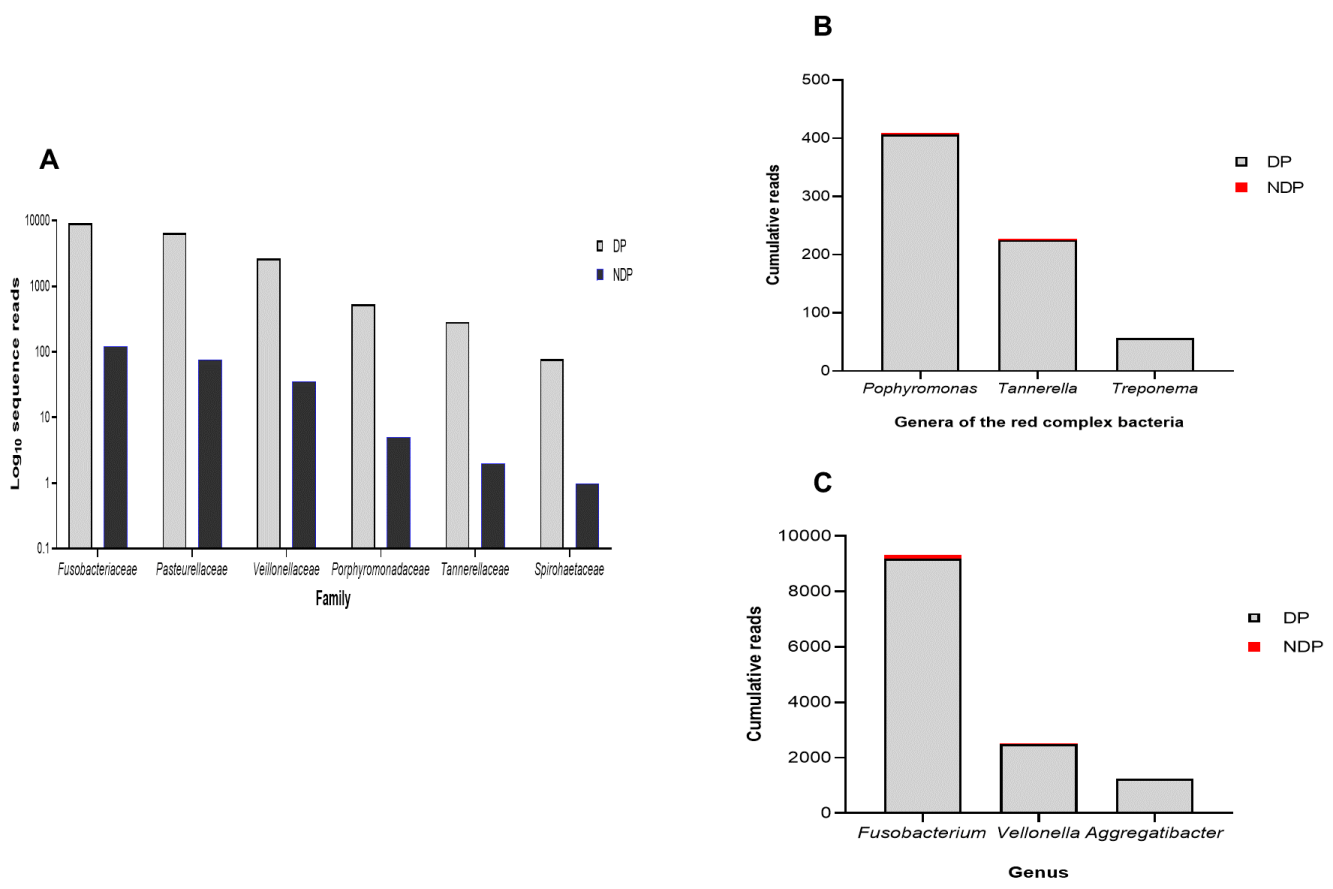
Subgingival microbiota of diabetic (DP) and non-diabetic (NDP) patients. Relative abundance of ( **A**) six bacterial families; ( **B**) genera of the red complex bacteria; ( **C**) genera of selected non-red complex bacteria.

Furthermore, as shown in Figure 1C and D, the subgingival microbiota profiles observed at the families level were relatively similar between DP and NDP groups, indicating that the predominant cooperative network microbiome is still conserved . However, when the six targeted bacteria were analysed at family level, the DP and DP groups' bacterial profiles were predominantly by *Pasteurellaceae*, followed by *Veillonellaceae, Porphyromonadaceae, Tannerellaceae*, and *Spirochaetaceae*. All bacteria belonging to these family were increased in the DP group compared with the NDP group ( Figure 2A). Therefore, the obtained length-sequences were further analysed to determine if diabetes, which alters the nature of inflammatory response ^25^, also influenced the relative abundance of six genera of periodontal pathogens selected in this study. 

### Distribution of six subgingival microbial at genus and species level

In this study, we focused to on identifying the red complex ( *P. gingivalis, T. denticola, and T. forsythia*), which a lot of studies have described as the most important pathogens in adult periodontal disease ^26, 27^. We analysed the red complex at genus and species levels of the subgingival niche, as species identification is important because it provides information regarding periodontal disease's pathogenicity and a detailed description of the subgingival microbiome in a diabetic subject. We found that among the three genera belong to these species, the topmost prevalent genera were *Porphyromonas*, followed by *Tannerella*, and *Treponema* ( Figure 2B). As shown in Figure 2B, the cumulative reads belong to these genera were significantly increased in DP subjects compared with the NDP group, and more specifically, *Treponema* was only found in the DP group. This result might indicate that the different quantities of red complex bacteria are more likely due to host diabetic-related responses. However, as with other systemic factors, there are very diverse clinical and medical parameters that might affect the composition of the oral microbiome in systemic disorder patients ^28^. Hence, it is more likely that the red complex bacteria in subgingival plaque microbiome observed in periodontitis subject was affected by several concurrent factors, which we did not include in this study.

In addition to the red complex bacteria differences observed between DP and NDP subjects, we studied the differences in the individual microbial genera belong to *Fusobacteria, Veillonella*, and *Aggregatibacter*. In general, we found that the most abundant bacteria in both subject groups (DP and NDP) belonged to *Fusobacterium sp*. ( Figure 2C). 

**Figure 3.**
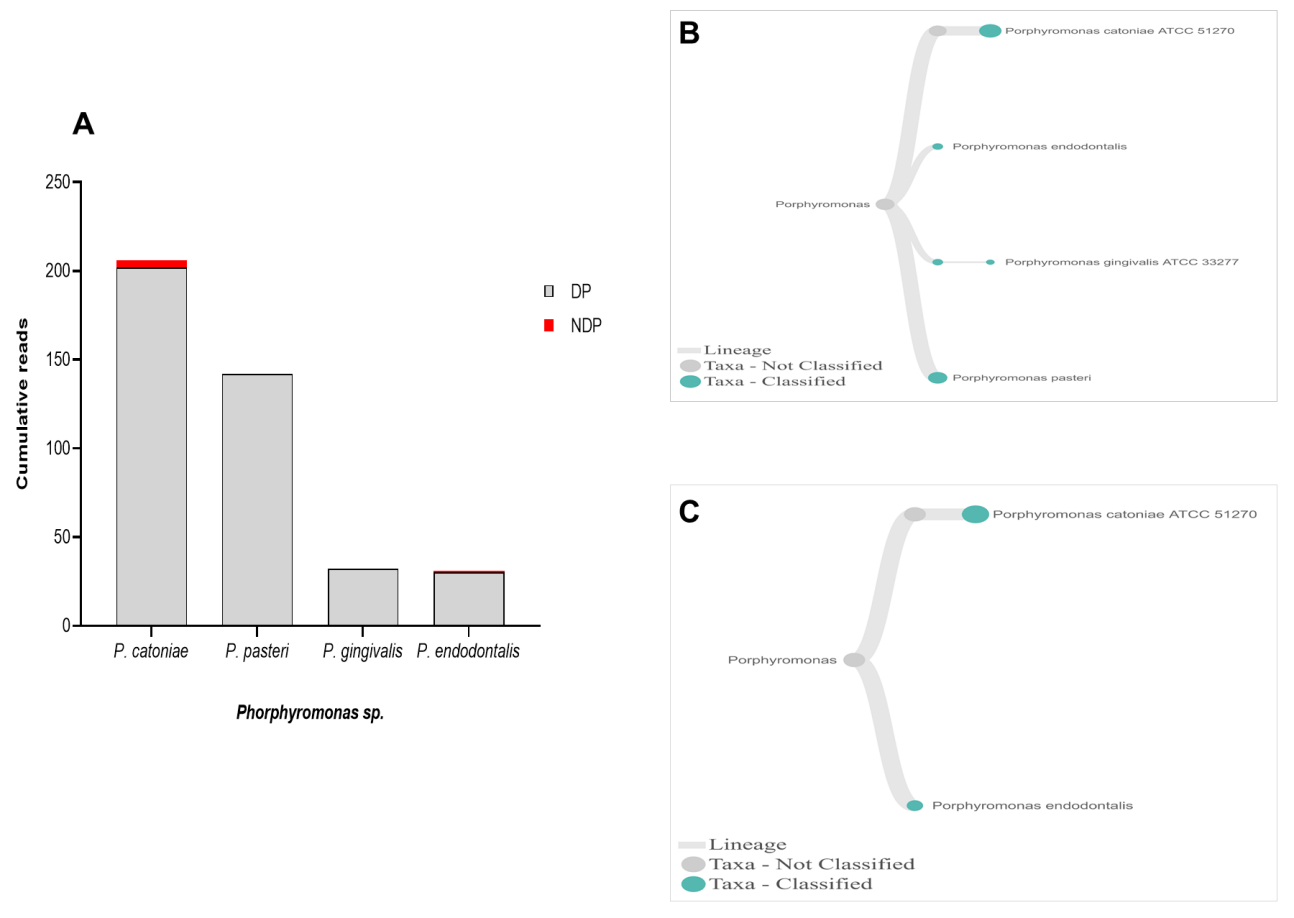
*Porphyromonas sp.* in pooled subgingival microbiota samples obtained from diabetic (DP) and non-diabetic (NDP) patients. ( **A**) Abundance of *Porphyromonas* sp; ( **B** and **C**) dendrograms showing the variability of *Porphyromonas sp*. in DP ( **B**) and NDP ( **C**) groups.

**Figure 4.**
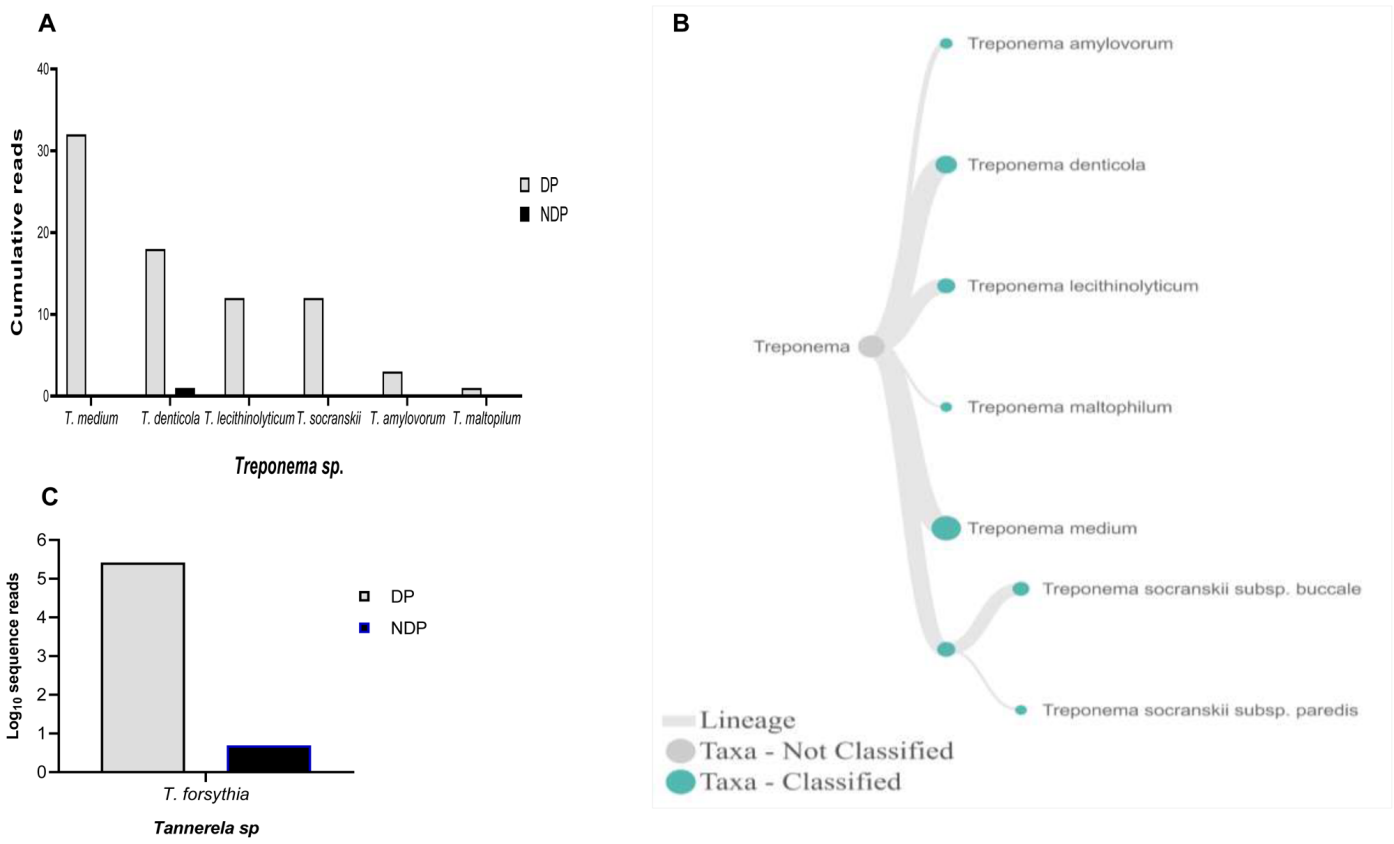
*Treponema* and *Tannerella sp.* in pooled subgingival microbiota samples obtained from diabetic (DP) and non-diabetic (NDP) patients. Relative abundance of ( **A**) six *Treponema * sp; and ( **B**) Dendogram showing the variability of *Treponema* sp., across pooled samples. ( **C**) *Tannerella forsythia*.

When analysis was focused to the genus *Phorphyromonas*, our data indicated that these genera, where its sequences were abundant in the DP group, comprise mainly species *P. catoniae*, followed by *P. pasteri*, *P. gingivalis*, and *P. endodontalis*, while only *P. catoniae* and *P. endodontalis* sequences was detected in NDP group ( Figure 3A–C). For *Treponems*, which are typically restricted to the subgingival crevice ^29^, the full length of 16S RNA gene belonging to this genus were found in six species and one subspecies in DP group, i.e. *T. medium, T. denticola, T. lecithinolyticum, T. maltophilum, T. amylovorum,* and *T. socranskii* as well as it subspecies ( Figure 4A and B). Lastly, sequences identified as *Tannerella* was *Tannerella forsythia*, which was more abundant in the DP than in the NDP group ( Figure 4C). However, by comparing to *P. gingivalis* and *T. denticola*, the cumulative reads of *T. forsythia* were found to be higher (not shown), suggesting that individuals with diabetes may have an increase in the subgingival abundance of the *T. forsythia*,

When species analysis was focused on *P. gingivalis*, literature shows that this species has been proposed as an important keystone pathogen-induced dysbiosis in periodontitis conditions ^30^. It has the ability to modify the oral microbiota composition ^31^. In this study, the bacterium was only found in a sample collected from DP patients, while we obtained all the samples from chronic periodontitis patients. Additionally, in the EPI2ME 16S workflow, nanopore sequence reads are blasted against the NCBI database for 16S DNA. Although it is possible that certain species are not represented in the database, this was not the case for *P. gingivalis*, as its 16S rRNA gene sequence can be retrieved from NCBI refSeq database. Thus, our study contradicts the previous report showing that *P. gingivalis* was associated with periodontitis in patients without diabetes ^32^.

As also shown in literature, the presence of red complex bacteria in subgingival niche are usually found with consortia, which include various species belong to the “orange, green, and purple complex” ^33, 34^ as well as non-pathogenic microorganisms ^35^. Since this polymicrobial consortium comprising the mix species induced significant increased alveolar bone resorption than the mono species ^36^, our result may suggest the difference in host response between the DP and NDP groups, and we did not explore this in this study. Furthermore, our results are in line with a previous study that recovered several periodontal pathogens, including *A. actinomycetemcomitans*, *Campylobacter rectus, F. nucleatum*, and *P. intermedia*, which was similar in both diabetic and non-diabetic subjects, but *P. gingivalis* was more frequently detected in individuals with diabetes ^37^. Our finding is also consistent with previous reports, in which *P. gingivalis* is a quantitatively minor constituent of biofilms associated human periodontitis ^38– 40^, in addition to its association with progressive bone loss in periodontitis patients ^41^, particularly those with diabetes ^40^.

Other studies showed that the red complex species can be detected in higher numbers when the disease reaches the advanced state ^8^. However, this study showed that only the read counts of *T. forsythia* were found higher in patients with diabetes than the other red complex bacteria species. Our result supports the idea of polymicrobial synergy and dysbiosis for periodontitis, which highlights the importance of other bacterial species in keystone pathogenesis ^42^. Thus, species other than the red complex species may have similar keystone role in periodontitis ^30^, as shown in this study. Another interesting finding was that we observed *T. forsythia* to be associated with periodontitis, and it did not relate to diabetes as its DNA was detected in all samples obtained from DP and NDP, and to lesser extent was the DNA of *P. endodontalis*. Similar findings have been reported previously ^43, 44^. Similarly, *P. catoniae*, which has been found in the mouth of infants before eruption of their teeth ^45^, was detected in both DP and NDP groups in our study.

In this study, we observed that although the presence of the red complex species in DP group had similar trends as was seen in NDP group, two of them ( *T. denticola* and *T. forsythia*) showed differences in abundance. This result might indicate that the different quantity is more likely due to host diabetic-related response. However, there are very diverse clinical and medical parameters that might affect the composition of the oral microbiome in systemic disorder patients ^28^. Hence, it is more likely that the subgingival plaque microbiome observed in this study was affected by several concurrent factors.

### Analysis of *Aggregatibacter, Fusobacterium*, and *Veillonella*

In addition to the red complex bacteria differences observed between DP and NDP subjects, we studied the differences in the individual microbial species belong to *Aggregatibacter, Fusobacteria*, and *Veillonella.*

**Figure 5.**
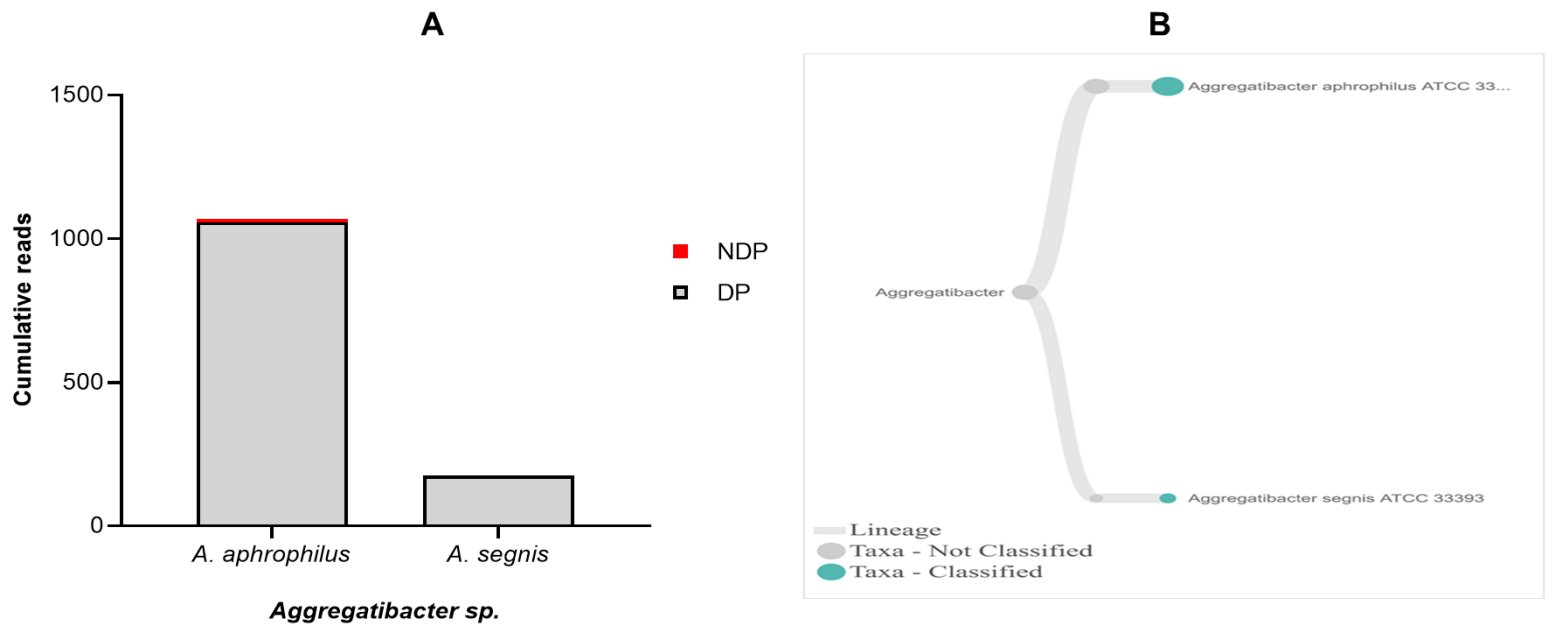
*Aggregatibacter sp.* in pooled subgingival microbiota samples obtained from diabetic (DP) and non-diabetic (NDP) patients. ( **A**) Abundance of *Aggregatibacter sp.*; ( **B**) dendrograms showing the same *Aggregatibacter sp.* across pooled samples.

Regarding *Aggregatibacter sp*., *A. actinomycetemcomitans* have been officially designated as aetiology agents of periodontitis, together with *P. gingivalis*, and *T. forsythia* ^46, 47^. Hence, our aim was to find out the presence of *A. actinomycetemcomitans* in a sample obtained from DP and NDP groups. While the DNA sequence of *A. acitnomycetemcomitans* was not present at any samples tested, we did find *A. aphrophilus* and *A. segnis*. These two species have been known to belong to the genus of *Aggregatibacter*, in addition to *A. actinomycetemcomitans* ^48^. Therefore, this finding is the first step towards understanding the potential contribution and a partnership between *A. aphrophilus* and *A. segnis* with *P. gingivalis*, and *T. forsythia* in periodontitis patients with and without diabetes. Comparison of the cumulative reads of the two species ( *A. aphrophilus* and *A. segnis*), between DP and NDP groups is shown in Figure 5A and B. Additionally, despite the presence of *A. aphrophilus* and *A. segnis*, our data are consistent with a previous report that species belonging to genus *Aggregatibacter* were present at a relatively low level compared to other periodontal pathogenic species ^49^. Another study also showed that both *A. actinomycetemcomitans* and *Prevotella intermedia* are of only minor importance in periodontal disease progression ^50^.

**Figure 6.**
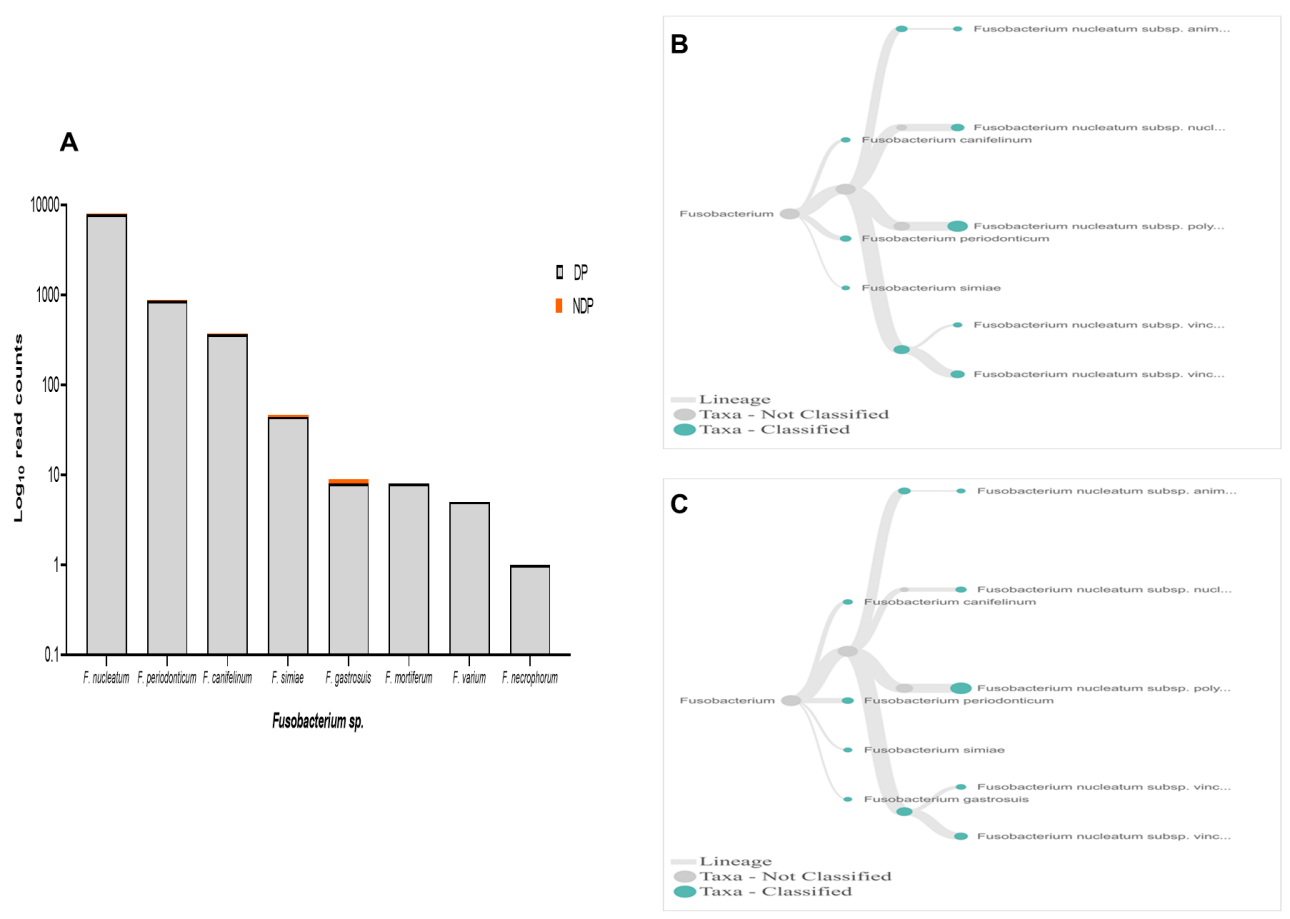
*Fusobacterium sp.* in pooled subgingival microbiota samples obtained from diabetic (DP) and non-diabetic (NDP) patients. ( **A**) Abundance of *Fusobacterium sp*.; ( **B** and **C**) dendrograms showing the variability of *Fusobacterium sp*. in DP ( **B**) and NDP ( **C**) groups.

In terms of *Fusobacteria*, within oral cavity *F. nucleatum* is the most abundant species, in both diseased and healthy individuals ^51, 52^. This species has a role in the progression of periodontal disease due to its ability to build a physical relationship (co-aggregation) with other oral bacterial species, notably with *P. gingivalis* and *T. denticola* formation of biofilm ^53^. Also, in the subgingival model, the count of *P. gingivalis* and some tested bacteria significantly decreased in the presence of *Fusobacterium sp*./spp. ^54^. Our data showed that, although the species variability of *Fusobacterium sp*. was relatively similar between the two groups tested, the cumulative reads of *F. nucleatum* was found more abundant in the DP group ( Figure 6A–C). In contrast, the reverse was found for *P. gingivalis* ( Figure 3A). Hence, it is important to carry out studies that evaluate the possibility of host response-associated diabetes regulating the interaction between *F. nucleatum* and *P. gingivalis*.

**Figure 7.**
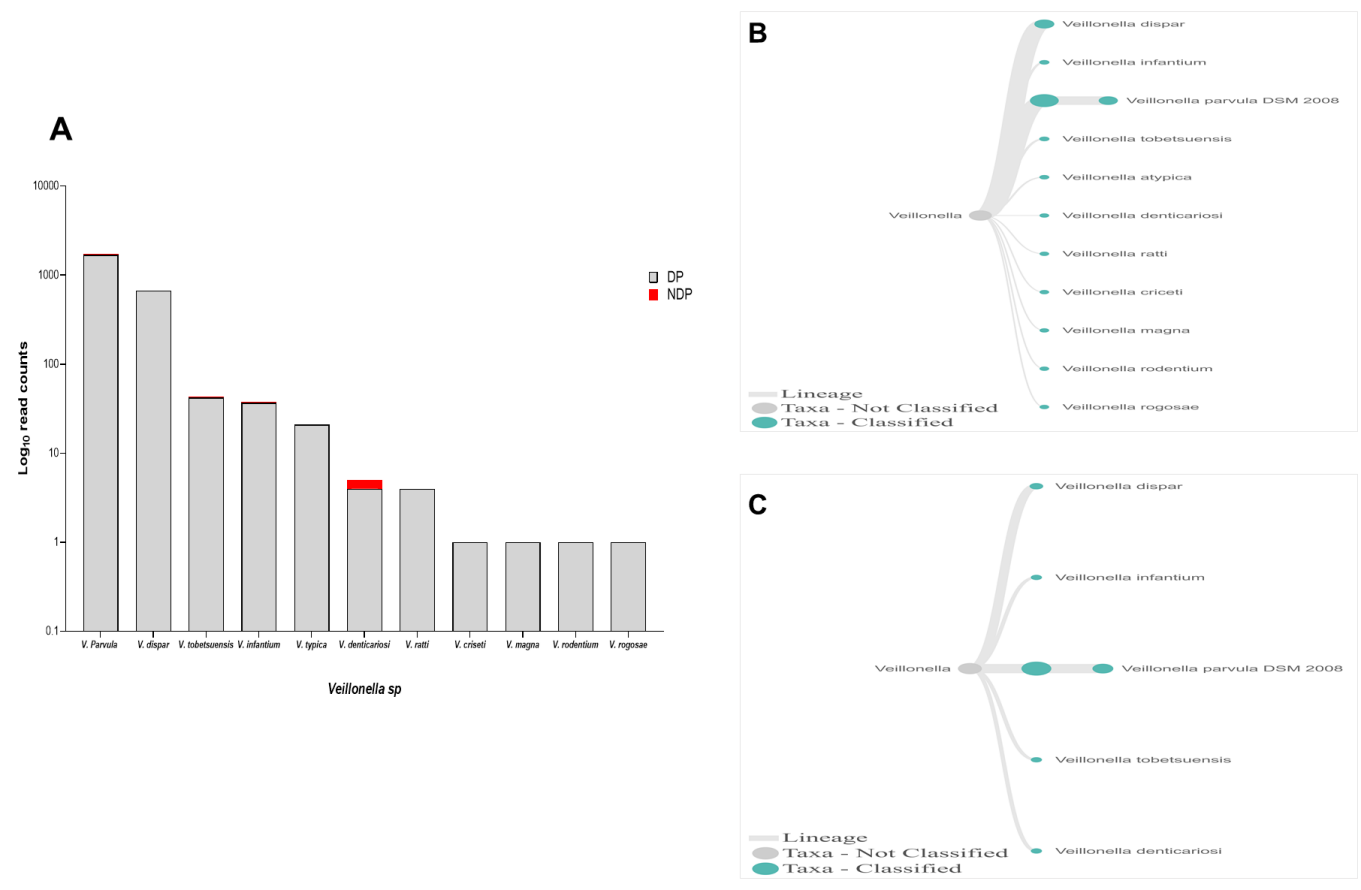
*Veillonella sp.* in pooled subgingival microbiota samples obtained from diabetic (DP) and non-diabetic (NDP) patients. ( **A**) Abundance of *Veillonella sp*.; ( **B** and **C**) dendrograms showing the variability of *Veillonella sp*. in DP ( **B**) and NDP ( **C**) groups.

In this study, Firmicutes bacteria at genus level to be analysed was *Veillonella sp*. We found that the annotation accuracy for *Veillonella* at the genus level was 88%. The cumulative reads of sequences belonging to *Veillonella sp*. consisted of eleven and five species in DP and NDP, respectively ( Figure 7A–C). We found that *V. parvula* was the predominant Firmicutes bacteria in subgingival microbiota of both groups, with more abundance in the DP group. Additionally, the results of this study was similar with a previous report elsewhere, in which *V. rogosae* was detected at a low number in DP patients ^55^, and was not detected in NDP individuals. Although it had been proposed to be used as an index for the state of chronic periodontitis ^55^, there is no clear explanation at present regarding the increased number of *V. parvula* in subgingival biofilms of diabetic patients. Our result, however, may indicate different environment conditions due to diabetes that my lead to increased number of *V. parvula* in subgingival niche. Interestingly, *Veillonella sp*. have been reported to have the ability to inhibit the host-cell effect of *P. gingivalis* ^56^, the red complex species that we found in lower abundance in the subgingival niche of DP patients in the current study. Thus, the difference in the amount and other bacterial species is not sufficient to explain the difference in periodontitis severity in a patient with diabetes. Although the host's immunological response may be influenced by diabetes ^25^, in the case of our subjects, other risk factors, including genetic background ^57^ may also affect inflammation and periodontal disease expression ^58^, which we did not include in this study. Considering these facts, we suggest that in periodontitis patient with diabetic, the subgingival microbiota formed by a low level of red complex and other representative bacteria may indicate that the red complex bacteria are necessary but insufficient to be linked to diabetes.

There are some limitations of this study. First, we compared the subgingival microbiome profile based on pooled PCR amplicons separated by the DP and NDP groups, respectively, not with health and disease sites as controls. It is also well known that detailed site-specific information might be lost when using pooled samples for microbial analysis ^59^. Although being inferior to the non-pooling sample, this study suggests the pooling approach for sequencing studies, particularly if there are budgetary constraints that do not permit individual sample runs' analytical execution. Lastly, the descriptive analysis prevented us from testing the directional relationship between diabetes and periodontitis.

## Conclusion

This is the first study in Indonesia to show that using the Nanopore MinION sequencing technology, we can investigate the presence of a consortium of red complex bacteria ( *P. gingivalis, T. forsythia,* and *T. denticola*) that includes three genera ( *Aggregatibacter, Fusobacterium*, and *Veillonella*) in periodontitis subjects with and without diabetes. The present study revealed that the abundance of the sequence reads of six selected bacteria in subgingival microbiome were strongly affected by diabetic condition. All sequences observed in a large number were derived from the DP group. However, the six selected periodontal pathogens profile was relatively similar between DP and NDP pooled DNA samples. Therefore, we reject the hypothesis that the composition of subgingival biofilm in DP patients is more variable than in periodontitis subjects without diabetes. Additionally, one species belonging to the red complex bacteria ( *P. gingivalis*) was only found in the subgingival microbiome of DP. Lastly, the capability of differentiating bacterial species, and even subspecies, as shown in this study, makes the MinION sequencer useful for pathogen detection in periodontitis subjects since it enables full-length 16S rRNA amplicon sequencing, while the reads can be analysed in real-time. However, we suggest, when investigating the subgingival microbiome of periodontitis patient with diabetes, there should be evidence in the presence of the targeted bacteria before the detection of attachment loss or bone loss

## Data availability

### Underlying data

Open Science Framework: A pilot study of red complex and three genera subgingival microbiome in periodontitis subjects with and without diabetes, evaluated by MinION, https://doi.org/10.17605/OSF.IO/DQE6F ^60^.

This project contains the following underlying data

- Subject data
- Fastq files

Data are available under the terms of the Creative Commons Zero "No rights reserved" data waiver (CC0 1.0 Public domain dedication).
