# Supplementary material for: A pilot study of red complex and three genera subgingival microbiome in periodontitis subjects with and without diabetes, evaluated by MinION platform
Source: F1000Res. 2021 Jul 12;10:79. Originally published 2021 Feb 8. [Version 4] doi: 10.12688/f1000research.28216.4 (PMC8261760; doi:10.12688/f1000research.28216.4)
Supplement: Peer review history

# Peer review: Reviewer response for version 4

**DOI:** 10.5256/f1000research.58480.r89415

**Published:** 2021-07-13

**Authors:** Heni Susilowati

**Affiliations:** 1 Oral Biology Department, Universitas Gadjah Mada, Yogyakarta, Indonesia

I have approved the revision of the methods section so that the flow of research methods becomes easier to understand.

Is the work clearly and accurately presented and does it cite the current literature?

Yes

If applicable, is the statistical analysis and its interpretation appropriate?

Not applicable

Are all the source data underlying the results available to ensure full reproducibility?

Yes

Is the study design appropriate and is the work technically sound?

Yes

Are the conclusions drawn adequately supported by the results?

Yes

Are sufficient details of methods and analysis provided to allow replication by others?

Yes

Reviewer Expertise:

Oral Microbiology and Immunology

I confirm that I have read this submission and believe that I have an appropriate level of expertise to confirm that it is of an acceptable scientific standard.

# Peer review: Reviewer response for version 4

**DOI:** 10.5256/f1000research.58480.r89416

**Published:** 2021-07-13

**Authors:** Wan Izlina Binti Wan Ibrahim

**Affiliations:** 1 Department of Oral and Craniofacial Sciences, Faculty of Dentistry, Universiti Malaya, Kuala Lumpur, Malaysia

I have no further comments to make.

Is the work clearly and accurately presented and does it cite the current literature?

Yes

If applicable, is the statistical analysis and its interpretation appropriate?

Not applicable

Are all the source data underlying the results available to ensure full reproducibility?

Yes

Is the study design appropriate and is the work technically sound?

Yes

Are the conclusions drawn adequately supported by the results?

Yes

Are sufficient details of methods and analysis provided to allow replication by others?

Yes

Reviewer Expertise:

Medical biochemistry and microbiology/ proteomics/metabolomics/human milk/oral biology

I confirm that I have read this submission and believe that I have an appropriate level of expertise to confirm that it is of an acceptable scientific standard.

# Peer review: Reviewer response for version 3

**DOI:** 10.5256/f1000research.58058.r88545

**Published:** 2021-07-06

**Authors:** Heni Susilowati

**Affiliations:** 1 Oral Biology Department, Universitas Gadjah Mada, Yogyakarta, Indonesia

Thank you for the response from the author to the previous suggestions. However, there are still similarities between the work described in paragraph 3 of the Methods with the beginning of paragraph 5.

 In addition, I suggest a description of the DNA isolation procedure in the Microbial Sampling section, following paragraph 5.

 Thank you.

Is the work clearly and accurately presented and does it cite the current literature?

Yes

If applicable, is the statistical analysis and its interpretation appropriate?

Not applicable

Are all the source data underlying the results available to ensure full reproducibility?

Yes

Is the study design appropriate and is the work technically sound?

Yes

Are the conclusions drawn adequately supported by the results?

Yes

Are sufficient details of methods and analysis provided to allow replication by others?

Yes

Reviewer Expertise:

Oral Microbiology and Immunology

I confirm that I have read this submission and believe that I have an appropriate level of expertise to confirm that it is of an acceptable scientific standard.

# Response

**Published:** 2021-07-06

**Authors:** Boy Muchlis Bachtiar

**Affiliations:** Universitas Indonesia, Indonesia

Dear Editor,

 I have read the Reviewer's suggestion, and I revised the paragraph in the method section as suggested by the Reviewer.

 Thank you

# Peer review: Reviewer response for version 2

**DOI:** 10.5256/f1000research.55175.r87054

**Published:** 2021-06-21

**Authors:** Heni Susilowati

**Affiliations:** 1 Oral Biology Department, Universitas Gadjah Mada, Yogyakarta, Indonesia

This research is well organized and benefits the development of microbiology and periodontology. The research background is well explained. Results are well presented and easy to understand. The analysis is comprehensive. The conclusions represent the answers to the research problems.

 For the Methods section: The sequence of the study in the form of sampling of subgingival plaque isolates and microbial samples, respectively, is written in paragraphs 3 and 6 of the Methods section, whether they are two stages of the same or different work. The order of explanation of these stages is a bit confusing. Paragraph 6 may be more appropriate to be written in paragraph 3.

Is the work clearly and accurately presented and does it cite the current literature?

Yes

If applicable, is the statistical analysis and its interpretation appropriate?

Not applicable

Are all the source data underlying the results available to ensure full reproducibility?

Yes

Is the study design appropriate and is the work technically sound?

Yes

Are the conclusions drawn adequately supported by the results?

Yes

Are sufficient details of methods and analysis provided to allow replication by others?

Yes

Reviewer Expertise:

Oral Microbiology and Immunology

I confirm that I have read this submission and believe that I have an appropriate level of expertise to confirm that it is of an acceptable scientific standard, however I have significant reservations, as outlined above.

# Response

**Published:** 2021-06-22

**Authors:** Boy Muchlis Bachtiar

**Affiliations:** Universitas Indonesia, Indonesia

We appreciate for the reviewer (two) comments and suggestion. Therefore, we have revised this manuscript accordingly. Thank you.

 The reviewer's comment is regarding the explanation of subgingival plaque sampling and microbial samples. We agree, the explanation is rather confusing. Because they are the same work, we put both the paragraph 3 and 6, and rewritten them together as paragraph 1 under subheading "microbial sample.

# Peer review: Reviewer response for version 2

**DOI:** 10.5256/f1000research.55175.r86513

**Published:** 2021-06-16

**Authors:** Wan Izlina Binti Wan Ibrahim

**Affiliations:** 1 Department of Oral and Craniofacial Sciences, Faculty of Dentistry, Universiti Malaya, Kuala Lumpur, Malaysia

I have no further comments to make.

Is the work clearly and accurately presented and does it cite the current literature?

Yes

If applicable, is the statistical analysis and its interpretation appropriate?

Not applicable

Are all the source data underlying the results available to ensure full reproducibility?

Yes

Is the study design appropriate and is the work technically sound?

Yes

Are the conclusions drawn adequately supported by the results?

Yes

Are sufficient details of methods and analysis provided to allow replication by others?

Yes

Reviewer Expertise:

Oral Biology (Oral Biochemistry, Oral Microbiology, Oral Histology, Oral Physiology, Oral Environment); Traditional Medicine and Natural Products (Free radical biochemistry & DNA damage); Biotechnology, Medical Biotechnology (Gel- & non-gel-based proteomics, liquid chromatography & mass spectrometry); Proteins (Proteomics & glycoproteomics - cancer, disease biomarkers, human and mammalian milk)

I confirm that I have read this submission and believe that I have an appropriate level of expertise to confirm that it is of an acceptable scientific standard.

# Peer review: Reviewer response for version 1

**DOI:** 10.5256/f1000research.31209.r79850

**Published:** 2021-03-02

**Authors:** Wan Izlina Binti Wan Ibrahim

**Affiliations:** 1 Department of Oral and Craniofacial Sciences, Faculty of Dentistry, Universiti Malaya, Kuala Lumpur, Malaysia

**General comments:**

- The manuscript presents interesting findings regarding the presence of red complex and three genera subgingival microbiome in periodontitis subjects with and without diabetes, using ONT MinION platform, which is one of the most recent sequencing technologies.
- While detailing the advantages of the platform, it would be great to also describe about the limitations or drawbacks of this technology, because the authors made quite a number of postulations, some can be confounding, based on literatures that used other technologies or techniques. This platform is known to be error prone, particularly in studying small or rare sequence differences.
- It would be also be really great if the authors consider to include a method for bacterial identification and quantification that is able to validate these results, perhaps a preliminary study, if there is any?
- Suggest proofread again to recheck incorrect sentence structure, missing preposition, as well as minor spelling and grammar mistakes. E.g. phylum are sometimes capitalized when it is not actually a proper noun.

**Specific comments:**

**Method**

Microbial assessment:

- Eppendorf is a brand. Should be written as 'a microcentrifuge tube' instead of "an Eppendorf tube".
- I suggest changing the subheading "Microbial assessment", to **'Microbial sample collection'.
- It is unclear what the authors meant by " *The presence at genus and species level of the red complex bacteria (Phorphyromonas gingivalis, Treponema denticola, and Tannerella forsythia), and three other selected genera (Aggregatibacter, Fusobacterium, and Veillonella) were determined in subgingival biofilm samples." - *How did the authors determine "the presence" of these bacteria? Or the authors are actually already referring to the MinION sequencing already? If yes, kindly consider revising the sentence.
- Third paragraph "MinIONTM" - The authors should be consistent, whether to use MinION with ^TM^ (superscript) or without, throughout the text. 

MinION sequencing and data analysis:

- "Polymerase Chain Reaction" is not a proper noun, so there is no need to capitalize each word.

**Results and discussion**

Read analysis:

- Second paragraph, third sentence *"By comparing the read count, we found that the classified sequence reads in the pooled sample of DP were 112.173 and 1988, respectively, while in the pooled sample of NDP were 1478 and 172, respectively (Figure 1A)"*. Consider revising this sentence.
- Perhaps the authors intended to write 'we found that the classified and unclassified sequence reads in the pooled sample of DP were 112.173 and 1988, respectively, while the classified and unclassified sequence reads in the pooled sample of NDP were 1478 and 172, respectively.'?

Distribution of the subgingival microbiota at the phylum and family level

- Second paragraph, *"All bacteria belonging to these phyla were increased in the DP group compared with the NDP group (Figure 2A)."*. I believe 'phyla' should be replaced with 'families'.

Analysis of *Aggregatibacter, Fusobacterium*, and *Veillonella*

- Figure 5 - In the text, the authors described Figure 5A and 5B as showing the cumulative reads of *A. aphrophilus* and *A. segnis* between DP and NDP groups. However, the legend says (A) Abundance of Aggregatibacter sp.; (B) Dendogram showing the variability of Treponema sp., across pooled samples. (C) Tannerella forsythia. Kindly revise the legend. 

Is the work clearly and accurately presented and does it cite the current literature?

Yes

If applicable, is the statistical analysis and its interpretation appropriate?

Not applicable

Are all the source data underlying the results available to ensure full reproducibility?

Yes

Is the study design appropriate and is the work technically sound?

Yes

Are the conclusions drawn adequately supported by the results?

Yes

Are sufficient details of methods and analysis provided to allow replication by others?

Yes

Reviewer Expertise:

Oral Biology (Oral Biochemistry, Oral Microbiology, Oral Histology, Oral Physiology, Oral Environment); Traditional Medicine and Natural Products (Free radical biochemistry & DNA damage); Biotechnology, Medical Biotechnology (Gel- & non-gel-based proteomics, liquid chromatography & mass spectrometry); Proteins (Proteomics & glycoproteomics - cancer, disease biomarkers, human and mammalian milk)

I confirm that I have read this submission and believe that I have an appropriate level of expertise to confirm that it is of an acceptable scientific standard.

# Response

**Published:** 2021-03-03

**Authors:** Boy Muchlis Bachtiar

**Affiliations:** Universitas Indonesia, Indonesia

**General comments**:

 We highly appreciate the reviewers’ insightful and helpful comments on our manuscript.

- According to the reviewer suggestions, we will carefully rewrite the incorrect sentence structure, missing preposition, as well as minor spelling and grammar mistakes. For example, as suggested, the typo of “Phylum” will be been changed to 'phylum', in the new version of this manuscript.
- We also would like to correct some mistake we made in the original manuscript.
- In the new version of this manuscript, we will explain the limitations of the MinION platform, as well as adding the needed reference, and will put it in the last sentence of the second paragraph when explain the Synergistic phylum, as follows:

*"However, no genus belonging to Synergistic phylum was detected in this study. We assumed this is because of the low-read accuracy by MinION platform (Laver 2015), which complicates our complex samples' analysis."*

- Regarding the other identification and quantification method to validate this sequencing platform, our responce is we did not include bacterial identification and quantification method, such as real time-PCR to validate the sequencing result, as our main purposed was to estimate microbial community diversity using MinION platform.

**Specific comments:**

**Method**

**Microbial assessment**:

-  As suggested, the word “Eppendorf tube" has been changed to "microcentrifuge tube". Thank you.
- The sub heading "Microbial assessment" has been changed to "Microbial sample". Thank you.
- As suggested, the sentence, “The presence at genus and species level of the red complex bacteria (Phorphyromonas gingivalis, Treponema denticola, and Tannerella forsythia), and three other selected genera (Aggregatibacter, Fusobacterium, and Veillonella) were determined in subgingival biofilm samples, will be changed in the new version. Thus, the new sentence become,” *in order to detect the The presence at genus and species level of the red complex bacteria (Phorphyromonas gingivalis, Treponema denticola, and Tannerella forsythia), and three other selected genera (Aggregatibacter, Fusobacterium, and Veillonella) were determined in subgingival biofilm samples*.
- According to the reviewer suggestion, we have changed the word MinIONTM, and only write it as MinION throughout the text. Thank you.
- Yes, we agree, we will rewrite the “Polymerase Chain Reaction” without the capital word. Thank you.

**C**. **Results and discussion**

**Read analysis**:

- ”By comparing the read count, we found that the classified sequence reads in the pooled sample of DP were 112.173 and 1988, respectively, while in the pooled sample of NDP were 1478 and 172, respectively (Figure 1A)". Consider revising this sentence.

**Author response:** Yes, thank you for this important correction. As suggested, we will change the wrong sentence, In the new version, the corrected sentence will become “ *By comparing the read* count, we found that the classified *and unclassified sequence reads in the pooled sample of DP were 112.173 and 1988, respectively, while the classified and unclassified sequence reads in the pooled sample of NDP were 1478 and 172, respectively”.*

- Under the subtitle; within the second paragraph, “Distribution of the subgingival microbiota at the phylum and family level”.

**Author response:** We agree with the suggestion. Hence the word phyla have been changed to be families. Thank you.

**​​​​Analysis of Aggregatibacter, Fusobacterium, and Veillonella:**

-  Figure 5 - In the text, the authors described Figure 5A and 5B as showing the cumulative reads of A. aphrophilus and A. segnis between DP and NDP groups. However, the legend says (A) Abundance of Aggregatibacter sp.; (B) Dendogram showing the variability of Treponema sp., across pooled samples. (C) Tannerella forsythia. Kindly revise the legend.

**Author response**: Thank you very much for this very important correction. Accordingly, in the new version, the legend will been revised.
